# A prospective population-based cohort study of lactation and cardiovascular disease mortality: the HUNT study

**DOI:** 10.1186/1471-2458-13-1070

**Published:** 2013-11-13

**Authors:** Tone Natland Fagerhaug, Siri Forsmo, Geir Wenberg Jacobsen, Kristian Midthjell, Lene Frost Andersen, Tom Ivar Lund Nilsen

**Affiliations:** 1 Department of Public Health and General Practice, Norwegian University of Science and Technology, PO Box 8904 MTFS, 7491 Trondheim, Norway; 2 Department of Public Health and General Practice, HUNT Research Centre, Forskningsveien 2, 7600 Levanger, Norway; 3 Department of Nutrition, University of Oslo, Postboks 1046, Blindern 0317, OSLO, Norway; 4 Department of Human Movement Science, Norwegian University of Science and Technology, Dragvoll 7491 Trondheim, Norway

**Keywords:** Breastfeeding, Cardiovascular death, Maternal health

## Abstract

**Background:**

Recent studies suggest that lactation has long-term effects on risk for cardiovascular disease in women, but the effects on cardiovascular mortality are less well known.

**Method:**

In a Norwegian population-based prospective cohort study, we studied the association of lifetime duration of lactation with cardiovascular mortality in 21,889 women aged 30 to 85 years who attended the second Nord-Trøndelag Health Survey (HUNT2) in 1995–1997. The cohort was followed for mortality through 2010 by a linkage with the Cause of Death Registry. Adjusted hazard ratios (HR) for death from all causes and cardiovascular disease were calculated using Cox regression.

**Results:**

During follow-up, 1,246 women died from cardiovascular disease. Parous women younger than 65 years who had never lactated had a higher cardiovascular mortality than the reference group of women who had lactated 24 months or more (HR 2.77, 95% confidence interval [CI]: 1.28, 5.99). There was some evidence of a U-shaped association, where women who reported lactating 7–12 months had a HR of 0.55 (95% CI: 0.27, 1.09). No clear associations were observed among women 65 years or older.

**Conclusions:**

Excess cardiovascular mortality rates were observed among parous women younger than 65 years who had never lactated. These findings support the hypothesis that lactation may have long-term influences on maternal cardiovascular health.

## Background

Cardiovascular disease is the most common cause of death in women in all European countries and accounts for 26% of deaths before the age of 65 [1]. Thus, it is important to identify behaviours that may modify women’s risk of cardiovascular disease. Lactation is unique to women and may affect maternal metabolic health. A growing number of studies have shown that lactation has both short- and long-term beneficial effects on maternal cardiovascular risk factors, such as lipids, blood pressure, insulin and glucose homeostasis, obesity and diabetes [2-11], and that it may reduce the risk of cardiovascular events [12].

Even if one might expect that the reported favourable effects of lactation on cardiovascular risk factors would reduce the risk of cardiovascular death, the evidence is sparse. A recent study among Chinese textile workers found a slightly increased risk of death from ischemic heart disease among parous women who had never breastfed, though no consistent trend was observed [13]. However, the findings may not be applicable to populations with other birth and breastfeeding characteristics.

Norway has one of the highest breastfeeding rates in Europe, with 36% of the infants being breastfed at twelve months [14], hence providing a population of women that is quite suitable for studying the health effects of lactation. Furthermore, linkage between high quality health registries and population-based health surveys, such as the national Cause of Death Registry and the Nord-Trøndelag Health Study (HUNT) [15], allows long-term mortality follow-up studies.

To our knowledge, no previous study has examined the association of lactation and cardiovascular mortality in a Western general population. The purpose of this study was to assess the long-term cardiovascular mortality according to lifetime lactation duration in a population-based cohort of more than 20,000 Norwegian women who were followed prospectively for 15 years.

## Methods

### Study population

The Nord-Trøndelag Health Study (HUNT) is a population-based health study aiming at the total adult population ≥20 years of age in the county of Nord-Trøndelag, Norway. The HUNT Study comprises a total of three separate surveys and covers the period 1984–2006. Our study is based on data collected in the second survey (HUNT2, 1995–97). A detailed description of the data collection and methods has been published elsewhere [15]. Briefly, the current survey included two self-administered questionnaires and a clinical examination. The first questionnaire was sent by mail along with an invitation to a physical examination. The questionnaire included questions about health and lifestyle. The participants were requested to bring the completed questionnaire to the physical examination which included standardized measurements of height, weight, waist and hip circumferences, and blood pressure. Non-fasting concentrations of serum lipids were also measured. A second, more detailed questionnaire was handed out after the examination, and should be completed at home and returned by mail. Females were asked to provide data on reproductive history including number of births and corresponding lactation duration, as well as diseases and ailments, medical treatment, lifestyle, and socio-economic factors.

Among 47,312 women who were invited to HUNT2, a total of 35,280 (75.5%) women participated. For the purpose of this study, reasons for exclusions were: non-response to the second questionnaire (n = 4,957); current pregnancy (n = 575); age above 85 years (n = 309) or below 30 (n = 3,713); non-attendance at the clinical examination (n = 380); self-report of myocardial infarction (n = 1), stroke (n = 5) angina pectoris (n = 2) or diabetes (n = 31) prior to the first birth; unknown parity (n = 139); less than one year since their last child birth (n = 89); and unknown lactation history (n = 3,121). This left 21,889 women as eligible for statistical analysis, of whom 20,007 women reported to have given birth to at least one child.

### Lactation history

The second questionnaire included a self-reported lactation history. For each birth, the women reported year of birth and corresponding lactation duration in whole months (*“How many months did you breastfeed?”*). Lifetime duration of lactation was the sum of lactation duration for all births and was categorised into five levels (none, 1–6, 7–12, 13–23, and ≥24 months). The same categorization scheme was used in a previous study on lactation and long-term maternal metabolic health [11].

### Mortality end points

The unique 11-digit personal identification number of every Norwegian citizen enabled linkage of data from the HUNT2 survey with information from the Cause of Death Registry at Statistics Norway. Death reports are issued by physicians and public health officers and sent to the Cause of Death Registry. The reporting is mandatory by law. Deaths were classified according to the International Classification of Disease (ICD-9 and ICD-10), and cardiovascular disease was defined by ICD-9: 390 – 459 and ICD-10: I00 – 99. We calculated individual person time at risk from the date of participation in the HUNT2 Survey as baseline and until date of death from cardiovascular disease or from other causes, or until the end of follow-up at December 31st 2010, whichever occurred first.

### Clinical measurements

Height was measured without shoes to the nearest 1.0 cm and weight wearing light clothing to the nearest 0.5 kg. Body mass index was calculated as weight (kg) divided by the squared value of height (m^2^). Waist and hip circumferences were measured with a flexible steel band with the participants standing upright, and the numbers were rounded to the nearest 1.0 cm. Waist circumference was measured horizontally at the height of the umbilicus, and hip circumference was measured likewise at the thickest part of the hip [15].

Blood pressure was measured by specially trained nurses or technicians with oscillometric Dinamap 845 XT (Critikon, Tampa, FL) after adjustment of the cuff size according to the arm circumference. After an initial two minutes’ rest, the blood pressure was automatically measured three times at intervals of one minute [15]. In this study, we used the mean value of the second and third measurement of systolic and diastolic blood pressure.

The blood sample (non-fasting) drawn from all participants was centrifuged at the screening station and, on the same day, transported in a cooler to the laboratory. Serum lipids were analysed at the Central Laboratory, Levanger Hospital, using a Hitachi 911 Autoanalyser (Hitachi, Mito, Japan), applying reagents from Boehringer Mannheim (Mannheim, Germany). Total serum cholesterol and triglycerides were measured by an enzymatic colorimetric method. The day-to-day coefficients of variation were 1.3-1.9% for total cholesterol and 0.7-1.3% for triglycerides [15].

### Analyses

Baseline characteristics of the women were evaluated by Chi-square statistics and independent samples *t*-test. We used the following cut-off points and definitions: hypertension (blood pressure ≥ 140/90 mmHg or current antihypertensive treatment), elevated triglycerides (>1.7 mmol/L), diabetes (‘yes’ or ‘no’ to the question ‘*Do you have or have you had diabetes?*’ or blood glucose ≥ 11.1 mmol/l), obesity (body mass index ≥ 30 kg/m^2^), and abdominal obesity (waist circumference ≥88 cm).

Cox regression using attained age as the time-scale [16] was used to estimate hazard ratios (HR) and 95% confidence intervals (CIs) of death from all causes and from cardiovascular disease, first in three broad categories of nulliparous women and never/ever lactating women, and then more detailed in five categories of lifetime lactation duration among parous women. As previous studies have shown beneficial maternal metabolic effects with increasing duration of lactation [6,11,17,18], we used women with the longest lactation duration, i.e. ≥24 months, as a reference category. *P*-values from trend-tests were estimated among the parous women treating the categories of lactation duration as ordinal variables in tests for trends in the regression models.

To examine whether the effects of lactation duration were modified by age, we stratified the analyses according to baseline age (<65 years and ≥65 years). We also assessed statistical interaction using a likelihood ratio test of a product term of lactation duration and baseline age (<65 years and ≥65 years). Age 65 was chosen as the cut-off point since cardiovascular morbidity and mortality increases markedly after this age in women.

All analyses were adjusted for age in the time-scale. Potential confounders were *a priori* chosen based on established associations and/or plausible biological relations with the exposure of interest. Hence our multivariable adjusted models included the following: education (primary school, secondary school, college/university, and unknown), smoking status (current, former, and never smoker) hours of physical activity per week (no activity; < 3 hours light or < 1 hour hard activity; >3 hours light or 1 hour hard activity; >1 hour hard activity, and unknown), marital status (unmarried, divorced, widowed, and married/cohabiting), and parity (1, 2, 3 or ≥ 4 children).

Furthermore, we explored whether the association between lactation and cardiovascular mortality could be mediated by some conventional cardiovascular risk factors by adding body mass index, systolic and diastolic blood pressure, use of antihypertensive medication, triglycerides, total cholesterol, and diabetes to the fully adjusted model one at a time.

In supplementary analyses we restricted the sample to women aged 45 to 64 years in order to avoid misclassification of lactation due to changes in parity and thus lactation history after participation in HUNT2.

Departure from the proportional hazard assumptions was evaluated by Schoenfeld residuals [19]. All statistical tests were two-sided, and all analyses were performed using STATA software, release 11 for Windows (Stata Corp., College Station, Texas, USA).

### Ethical approval

The study was approved by the Regional Committee for Medical and Health Research Ethics and by the Norwegian Data Inspectorate. Written informed consent was given by all participants.

## Results

Baseline characteristics of the 21,889 women in our cohort are presented in Table 1. The majority of the 20,007 parous women (96.7%) had breastfed one or more children. Mean and median lifetime duration of lactation among women who had breastfed were 16.5 (SD 12.2) and 14.0 (Interquartile range [IQR] 14.0) months. There were some differences in parity and lactation habits between women younger than 65 years and women 65 years or older (Table 2). There were more nulliparous women in the oldest age group. On the other hand, older women were more likely to have four or more births, and to report lifetime duration of lactation ≥ 24 months compared to the younger women.

**Table 1:** Baseline characteristics of women in the HUNT2 Survey, Norway, 1995–97 (n = 21,889)

**Variables**^**a**^	**Ever lactated**	**Never lactated**	**Nulliparous**
	**(n = 19,350)**	**(n = 657)**	**(n = 1,882)**
Mean age, years (SD)	52.1 (14.1)	52.5 (12.5)	55.9 (16.8)
Age at delivery of first child, yrs	23.4 (4.1)	24.4 (5.2)	-
Lifetime lactation duration, months, median (IQR)^b^	14 (14)	-	-
Systolic blood pressure, mmHg, mean (SD)	135.9 (23.0)	139.5 (22.4)	141.6(25.1)
Diastolic blood pressure, mmHg, mean (SD)	79.5 (11.9)	82.1 (11.9)	81.1 (12.4)
Cholesterol, mmol/L, mean (SD)	6.0 (1.3)	6.2 (1.1)	6.2 (1.4)
Triglycerides, mmol/l, mean (SD)	1.6 (0.99)	1.7 (0.98)	1.6 (1.0)
Body mass index, kg/m^2^, mean (SD)	26.4 (4.5)	27.6 (4.9)	26.6 (5.0)
Waist –hip ratio, mean (SD)	0.80 (0.06)	0.81 (0.06)	0.80 (0.06)
Parity,			
Para 1, n (%)	1,793 (9.3)	175 (26.6)	-
Para 2, n (%)	7,305 (37.4)	260 (39.6)	-
Para 3, n (%)	6,187 (32.0)	151 (23.0)	-
Para 4 or greater, n (%)	4,065 (21.0)	71 (10.8)	-
College/university education, n (%)^c^	3,659 (18.9)	86 (13.1)	409 (21.7)
Never smokers, n (%)	9,345 (48.6)	248 (37.8)	1,085 (58.6)
High physical activity^d,e^, n (%)	3,468 (17.9)	86 (13.1)	311 (16.5)
Unmarried/divorced, n (%)	3,081 (15.9)	133 (20.2)	972 (51.8)
Cardiovascular disease^f^, n (%)	1,070 (5.5)	38 (5.8)	142 (7.6)
Hypertension^g^, n (%)	7.935 (41.0)	326 (49.6)	953 (50.6)
Elevated triglycerides^f^, n (%)	6,286 (32.5)	257 (39.2)	612 (32.4)
Diabetes, n (%)	523 (2.7)	31 (4.7)	78 (4.1)
Obesity, n (%)	3,655 (18.9)	186 (28.3)	406 (21.6)
Abdominal obesity^g^, n (%)	5,369 (27.8)	257 (39.2)	512 (27.3)

*Abbreviations:HUNT2* The second Nord-Trøndelag Health Survey, *SD* standard deviation.

^a^Continuous variables presented as mean (SD), categorical variables presented as number (%) in each category of lactation.

^b^Among women who had lactated (n = 19 350).

^c^Number of women in the category “unknown” in the total sample of women: n = 781 (ever lactated), n = 30 (never lactated), and n = 157 (nulliparous).

^d^High physical activity defined as ≥1 hour hard physical activity per week.

^e^Number of women in the category “unknown ”in the total sample of women: n = 1,976 (ever lactated), n = 75 (never lactated), and n = 309 (nulliparous).

^f^Cardiovascular disease = history of angina, stroke or myocardial infarction.

^g^Hypertension defined as bloodpressre ≥ 140/90 mmHg or the use of antihypertensive medication.

^f^Elevated triglycerides defined as triglycerides >1.7 mmol/L.

^g^Abdominal obesity defined as waist circumference ≥88 cm.

**Table 2:** Distribution of parity and lifetime lactation duration among women <65 years and women ≥65 years in the HUNT2 Survey, Norway, 1995–97 (n = 21,889)

**Variables**	**Women < 65 years**	**Women ≥ 65 years**
	**(n = 16,672)**	**(n = 5,217)**
**Parity**		
Nulliparous, n (%)	1,172 (7.0)	710 (13.6)
Para 1, n (%)	1,546 (9.3)	422 (8.1)
Para 2, n (%)	6,329 (38.0)	1,236 (23.7)
Para 3, n (%)	5,125 (30.7)	1,213 (23.3)
Para 4, n (%)	2,500 (15.0)	1,636 (31.4)
**Lactation**	(n = 15,500)	(n = 4,507)
No lactation	532 (3.4)	125 (2.8)
1 – 6 months, n (%)	3,286 (21.2)	699 (15.5)
7 – 12 months, n (%)	3,951 (25.5)	981 (21.8)
13 – 24 months, n (%)	4,662 (30.1)	1,278 (28.4)
≥ 24 months, n (%)	3,069 (19.8)	1,424 (31.6)

*Abbreviations:HUNT2* The second Nord-Trøndelag Health Survey.

During a median follow-up of 14.5 years (297,972 person-years), a total of 3,130 (14.3%) women died and of these 1,246 (39.8%) died from cardiovascular disease. Among these women, there were 449 deaths from ischemic heart disease and 353 deaths from stroke.

There was evidence that the effect of lactation duration was modified by age at baseline (*P-*values for interaction: 0.008 for total death and 0.003 for death from cardiovascular disease). Thus, all analyses were stratified by age above or below 65 years.

Table 3 shows age-stratified analyses of all-cause and cardiovascular mortality for never and ever lactating women, as well as for nulliparous women. Among parous women younger than 65 years, those who reported never lactating had an adjusted HR of 2.86 (95% CI: 1.51, 5.39) for death from cardiovascular disease compared with women who had ever lactated. Restricting the sample to women aged 45 to 64 years strengthened the association (HR 3.15, 95% CI: 1.66, 6.00) (Additional file 1: Table S1). Among women 65 years and older, we found no clear association between lifetime lactation history and cardiovascular mortality.

**Table 3:** Hazard ratios and 95% CIs for all causes and cardiovascular disease mortality in nulliparous and parous women by two categories of lifetime lactation duration, stratified by age (n = 21,889)

	**No. of persons**	**Person-years**	**All causes**					**Cardiovascular disease**				
			**No. of deaths**	**Mortality rate**^***a***^	**Simple model**^***b***^	**Fully adjusted model**^***c***^		**No. of deaths**	**Mortality rate**^***a***^	**Simple model**^***b***^	**Fully adjusted model**^***c***^	
					**HR**	**HR**	**95% CI**			**HR**	**HR**	**95% CI**
**Women <65 years**												
***Total***	16,672	237,286	670	28				115	5			
Ever lactated	14,968	213,175	587	28	1.00	1.00	Referent	98	5	1.00	1.00	Referent
Never lactated	532	7,491	33	44	1.44	1.33	0.93, 1.89	11	15	2.85	2.86	1.51, 5.39
Nulliparous	1,172	16,620	50	30	1.16	0.96	0.69, 1.33	6	4	0.82	0.41	0.16, 1.04
**Women ≥65 years**												
***Total***	5,217	60,685	2,460	405				1,131	186			
Ever lactated	4,382	51,588	1,987	385	1.00	1.00	Referent	914	177	1.00	1.00	Referent
Never lactated	125	1,480	53	358	1.05	1.01	0.77, 1.33	26	176	1.14	1.11	0.77, 1.69
Nulliparous	710	7,617	420	551	1.24	1.20	1.06, 1.36	191	251	1.21	1.20	1.00, 1.44

*Abbreviations:HUNT2* The second Nord-Trøndelag Health Survey, *HR* hazard ratio, *CI* confidence interval.

^*a*^Incidence per 10,000 person-years.

^*b*^Adjusted for maternal age.

^*c*^Adjusted for age, smoking status, physical activity, education, marital status and parity.

The HUNT2 Survey, 1995-2010.

**Figure 1:**
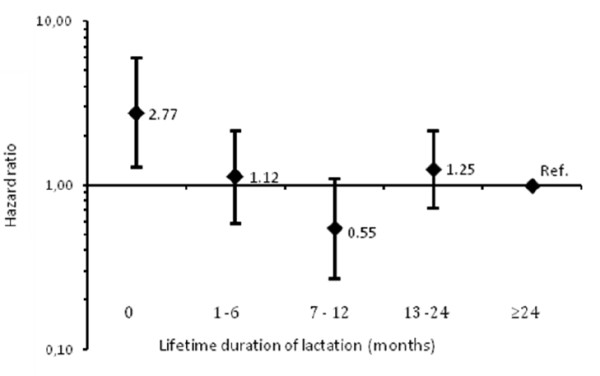
**Risk of death from cardiovascular disease associated with five categories of lifetime lactation duration for women <65 years (n = 15,500) with lifetime lactation duration ≥ 24 months as the reference category: Hazard ratios and 95% confidence intervals.** Adjusted for age, smoking status, physical activity, education, marital status and parity. Number of women in each category of lifetime duration of breastfeeding, with number of deaths in brackets, 0 months: n = 532 (11), 1–6 months: n = 3,286 (24), 7–12 months: n = 3,951 (15), 13–23 months: n = 4,662 (38), ≥ 24 months: n = 3,069 (21).

Among parous women younger than 65 years, we further explored the association across five categories of lactation duration (Figure 1). Approximately one in five women (22.5%) reported lifetime duration of lactation of 24 months or longer. Women who reported that they had never lactated had an adjusted HR of 2.77 (95% CI: 1.28, 5.99) for death from cardiovascular disease compared with women who reported ≥24 months of lifetime duration of lactation (Figure 1). We did not find a linear relationship across the categories of lifetime duration of lactation (*P*-trend 0.8). However, there was some evidence of a U-shaped association, where women who reported lactating 7–12 months had an adjusted HR of 0.55 (95% CI: 0.27, 1.09). In the restricted sample of parous women aged 45 to 64 years, those who reported no lactation had an adjusted HR of 3.03 (95% CI: 1.38, 6.70) while those who had lactated 7–12 months had an adjusted HR of 0.45 (95% CI: 0.21, 0.97) for death from cardiovascular disease compared with women who had lactated ≥24 months (Additional file 2: Figure S1).

Furthermore, we explored whether body mass index, systolic and diastolic blood pressure, use of antihypertensive medication, triglycerides and total cholesterol could mediate the association between lactation duration and cardiovascular mortality. The analyses showed that the excess mortality was attenuated after adjustment for these factors and that body mass index, systolic and diastolic blood pressure, use of antihypertensive medication and diabetes contributed to the attenuation (Table 4).

**Table 4:** **Hazard ratios of cardiovascular mortality by two categories of lifetime lactation duration (ever vs. never) in parous women < 65 years (n = 15,500)**^***a***^

**Adjustment for**	**HR (95% CI)**
Age, smoking status, physical activity, education, marital status and parity	2.86 (1.51, 5.39)
Age, smoking status, physical activity, education, marital status, parity +body mass index	2.78 (1.47, 5.27)
Age, smoking status, physical activity, education, marital status, parity, + systolic and diastolic blood pressure, and use of antihypertensive medication	2.76 (1.45, 5.22)
Age, smoking status, physical activity, education, marital status, parity, + triglycerides	2.92 (1.54, 5.51)
Age, smoking status, physical activity, education, marital status, parity, + total cholesterol	2.89 (1.53, 5.45)
Age, smoking status, physical activity, education, marital status, parity, + diabetes	2.72 (1.44, 5.13)
Age, smoking status, physical activity, education, marital status, parity, + body mass index, systolic and diastolic blood pressure, use of antihypertensive drugs, triglycerides, total cholesterol, and diabetes	2.63 (1.39, 4.99)

*Abbreviations:HUNT2* The second Nord-Trøndelag Health Survey, *HR* hazard ratio, *CI* confidence interval.

^*a*^Of 15,500 women, 109 died of cardiovascular disease.

The HUNT2 Survey, 1995-2010.

## Discussion

In this large study of 21,889 women with no report of cardiovascular disease prior to their first delivery, we found that cardiovascular mortality was almost three times higher in parous women younger than 65 years who had never lactated compared to women of the same age who reported lifetime lactation duration of ≥24 months. There was also some evidence of a U-shaped association, where women who reported lifetime duration of 7–12 months had almost half the risk of women who had lactated ≥24 months.

The present study adds further evidence that lactation is associated with long-term beneficial effects on the mothers’ metabolic health, and supports earlier reports that have shown an inverse relationship with visceral adiposity [10,20,21], hypertension [7], metabolic syndrome [4,18,22], type 2 diabetes [6,8,23], as well as other risk factors for cardiovascular disease [10,11,17] and myocardial infarction [12].

The only other study that has explored the association of lactation and cardiovascular mortality found results similar to ours [13]. This study from China suggested that parous women who did not breastfeed had a slightly increased risk of death from ischemic heart disease. By comparison, our study indicated a strong association between lactation and cardiovascular disease mortality. However, whereas the substantial sample size and large number of deaths in the Chinese study allowed the investigators to distinguish between different subgroups of cardiovascular mortality, this was not possible in the present one. Furthermore, it should be noted that the two studies differ in study design and sample characteristics. Whereas our study sample consisted of women from a general population in Central Norway, the Chinese study used textile workers in the largest city in the world. Thus, the study populations differed in age distribution, birth patterns, breastfeeding practice, disease distribution, longevity, health status, access to health care, level of education, and other socioeconomic and demographic variables.

Possible mechanisms that may explain our findings include several short- and long-term hormonal responses related to reproduction and lactation. Pregnancy induces significant changes in well-known risk factors for cardiovascular disease that could increase the risk of both cardiovascular morbidity and mortality. They include accumulation of central fat [24], increased blood pressure [25], an atherogenic lipid profile and insulin resistance [26], all of which may pose a risk to the mother’s health. Although the evidence regarding the effects of parity on maternal cardiovascular morbidity and mortality is conflicting [27,28], the hypothesis that lactation may affect the risk of metabolic disease by facilitating a faster resetting of the maternal metabolism after pregnancy has been proposed [29]. Our study indicates that the same hypothesis could be extended to include cardiovascular mortality as well.

We have previously reported an inverse and apparent dose-dependent association of lactation and body mass index, triglycerides, total and low density lipoprotein cholesterol, glucose, and blood pressure among women aged 50 years or younger in the current study population [11]. One explanation for these favourable effects of lactation could be that it drains calories as well as cholesterol from the lactating mother, thereby explaining the lower body mass index and lower cholesterol levels observed. Obesity [30] and dyslipidemia [31] are both considered independent risk factors for cardiovascular mortality, hence the cardiovascular mortality risk could be reduced by the favourable effect of lactation on the body mass index and cholesterol levels *per se*. The association between lactation duration and cardiovascular mortality in our study was slightly attenuated after adjustment for body mass, systolic and diastolic blood pressure, use of antihypertensive medication, and diabetes. This could suggest that the association may partly be mediated by these factors. Furthermore, a lower body mass index could also decrease serum lipids and blood pressure, and improve insulin and glucose homeostasis. A lower body mass index could thereby mediate the inverse association between lactation and lipids, blood pressure, diabetes risk, and ultimately with cardiovascular mortality.

However, given that both the amount and regional distribution [32,33] of fat mobilization seem to vary with time since delivery in lactating mothers, more complex mechanisms may be involved. It has been suggested that lactation induces alterations in the hypothalamic-pituitary-adrenal axis that could explain the increased mobilization of adipose tissue stores as well as influence what region of the body the fat is mobilized from [10].

The pituitary hormone oxytocin has been linked to the favourable short term effects of lactation on blood pressure [34]. Although circulating oxytocin has a very short half-life, even the long-term effects of lactation on blood pressure [22,35,36] have been attributed to oxytocin. Although the mechanisms are still unclear, it has been hypothesized that oxytocin induces a decrease in cortisol secretion and adrenocorticotropic hormone and an increase in central α_2_-adrenergic activity, which in turn may produce more lasting favourable effects on blood pressure [22,35].

Lactation may also influence several other pituitary hormones [37] and induce even long-term changes in the hypothalamic-pituitary axis [38]. A wide range of hormones may be involved in the mechanisms behind the association between lactation and cardiovascular mortality. Prolactin, vasopressin, estrogen, relaxine, progesterone, leptin, cortisol, thyroid-stimulating hormone and prolactin-inhibiting factor are either involved in, or influenced by, lactation or even produced by the mammary gland [39-42]. Prolactin has been associated with incident hypertension in post-menopausal women [43] and accelerated preclinical atherosclerosis [44]. Lactation was also associated with a decrease in prolactin levels in a prospective cohort study [45]. One may speculate that lactation triggers a complex hormonal cascade that induces both short-term and long-term effects on maternal metabolic health, making several hormones work together on a number of different factors like blood pressure regulation, lipolysis and lipogenesis, glucose homestasis and insulin secretion, lipid homeostasis, and inflammatory processes. This could occur as an interaction between reproductive and metabolic hormones, and potentially via metabolic pathways yet not unveiled.

We found an association between lactation and cardiovascular mortality only in women younger than 65 years. There are several possible explanations for this observation. The biological effect of lactation may wane with time, as other age-related cardiovascular risk factors become more prevalent. Moreover, other risk factors for cardiovascular disease, such as obesity [46] show stronger associations with mortality in younger than in older age groups, possibly reflecting either selective survival or inability to capture relative differences in mortality in older age groups [47]. Finally, more women in the oldest age group reported higher parity. One may speculate that if higher parity increases cardiovascular mortality, the beneficial effects of lactation may not be sufficient to outweigh the risk related to grand multiparity.

The strengths of our study include the large sample size, wide age range, high rates of both study participation and lactation, standardised measurements, and the long follow-up time from 1995–97 through 2010. Combined with the national end-point registry that provides reliable data on deaths, and the personal identification number allocated to each citizen, our study provides a unique opportunity to study the association between lactation duration and cardiovascular mortality.

Some limitations should be noted. First, women who breastfeed their children may possibly have better health, healthier lifestyles and higher socioeconomic status than those who do not [48], and therefore have lower risk of cardiovascular mortality. Breastfeeding rates are very high among Norwegian women, and fewer than 4% of our entire sample had never lactated. Thus, the women who had lactated and those who had not may have differed in terms of major confounders. However, adjustment for factors known to be associated both with lactation duration and risk of cardiovascular mortality did not materially change the estimated associations. In women younger than 65 years, we observed the lowest risk of cardiovascular mortality among those who reported lifetime duration of lactation of 7 – 12 months. This could reflect an optimal duration of lactation, although residual confounding by unmeasured and unknown factors, including diet and pre-pregnancy and early postpartum health status cannot be ruled out. Furthermore, lifetime duration of lactation is largely influenced by the number of childbirths, and thus it may be difficult to distinguish the effect of lactation from the effect of parity.

Additionally, we cannot rule out the issue of reverse causation*.* Obesity [49] and type 1 diabetes [50] have been linked to difficulties with initiating lactation as well as lactation duration. Furthermore, women with preeclampsia breastfed less than women without the condition [51], and reduced breastfeeding rates in the early postpartum period have been reported in women with polycystic ovary syndrome [52]. Hence, shorter lactation duration could be a marker for an already existing metabolic disease, which in turn could influence whether the women lactate and how long they did so. Unfortunately, with the exception of the women’s self reports of diabetes prior to the birth of their first child, we had no other data available on metabolic disorders prior to or during their pregnancies. The women who reported diabetes prior to their first child birth were excluded from our analyses, and reverse causation due to diabetes is therefore unlikely. Since pre-pregnancy measurements of weight and height were not available, we adjusted only for body mass index at study participation in supplementary analyses. That gave no substantial change in our estimates. It should be noted, however, that obesity may either precede [49] or follow lactation practices [10,53], and one may argue that body mass index measured at study participation would act as an intermediate factor.

Moreover, lactation was assessed retrospectively. Nevertheless, maternal recall of lactation seems to be fairly valid and reliable [54], even decades after weaning [55]. Indeed, in a population of Norwegian women breastfeeding duration was recalled quite accurately 20 years after mothers gave birth [56]. Even so, we cannot rule out the possibility of misclassification. Still, it is unlikely to be differential for cardiovascular risk factors. Our observed estimates are therefore likely to be conservative.

Lastly, during the follow-up of 15 years, the baseline variables parity and lactation history may have changed. Some of the women who reported no lactation at baseline might have given birth to another child and lactated during the follow-up period. If the associations between lactation and cardiovascular disease are causal, our estimates are most likely underestimations. This notion is supported by the observed strengthening of the estimates when we restricted the analyses to women whose parity and lactation history were unlikely to have changed during follow-up (i.e. aged 45–64 years).

## Conclusion

In this long-term follow-up, excess cardiovascular mortality rates were observed among parous women younger than 65 years who had never lactated, and the risk was lowest among women who reported lifetime duration of lactation of 7–12 months. Whether lactation acts as a marker of health, as a protective factor *per se*, or through complex hormonal interactions is unclear. Nevertheless, if substantiated, our findings might suggest the need for vigorously promoting these benefits of breastfeeding, especially in countries, where the rate of initiation is low and the breastfeeding duration is short. Future studies should include maternal metabolic factors before and during pregnancy that may affect both breastfeeding practices and later maternal cardiovascular health in order to elucidate the observed associations further.

## Abbreviations

CI: Confidence interval; HR: Hazard ratio; HUNT: The Nord-Trøndelag Health Study Norway; HUNT2: The second Nord-Trøndelag Health Survey; ICD-9: International statistical classification of diseases and related health problems ninth revision; ICD-10: International statistical classification of diseases and related health problems tenth revision.

## Competing interests

The authors declare that they have no competing interests.

## Authors’ contributions

Research fellow TNF, conceived the idea in the present study, conducted the analyses and is the principal author of the manuscript. Professor KM participated in the planning and data collection in the HUNT2 study. He also took part during the planning of the present study, the analyses and interpretation of the results, and the preparation of the manuscript. Professor TILN participated in the analyses, interpretation of the results, and preparation of the manuscript. Professor SF took part during the planning of the present study, the interpretation of the results, and the preparation of the manuscript. Professor LFA and Professor GWJ took part in the interpretation of the results, and the preparation of the manuscript. All authors read and approved the final manuscript.

## Pre-publication history

The pre-publication history for this paper can be accessed here:

http://www.biomedcentral.com/1471-2458/13/1070/prepub

## Supplementary Material

Additional file 1: Table S1 Hazard ratios and 95% CIs for all causes and cardiovascular disease mortality in nulliparous and parous women aged 45 to 64 years by two categories of lifetime lactation duration (n=8,477). The HUNT2 Survey, 1995-2010. Click here for file

Additional file 2: Figure S1 Risk of death from cardiovascular disease associated with five categories of lifetime lactation duration for parous women in the age group 45 – 64 years (n = 7,954) with lifetime lactation duration ≥ 24 months as the reference category: Hazard ratios and 95% confidence intervals. Adjusted for age, smoking status, physical activity, education, marital status and parity. Number of women in each category of lifetime duration of breastfeeding, with number of deaths in brackets, 0 months: n = 325 (11), 1–6 months: n = 1,838 (22), 7–12 months: n = 2,140 (11), 13–23 months: n = 2,268 (35), ≥ 24 months: n = 1,383 (19). Click here for file
