# Supplementary material for: A prospective population-based cohort study of lactation and cardiovascular disease mortality: the HUNT study
Source: BMC Public Health. 2013 Nov 13;13:1070. doi: 10.1186/1471-2458-13-1070 (PMC3840666; doi:10.1186/1471-2458-13-1070)
Supplement: Additional file 1: Table S1 — Hazard ratios and 95% CIs for all causes and cardiovascular disease mortality in nulliparous and parous women aged 45 to 64 years by two categories of lifetime lactation duration (n=8,477). The HUNT2 Survey, 1995-2010. [file 1471-2458-13-1070-S1.doc]

**Table I.** Hazard ratios and 95% CIs for all causes and cardiovascular disease mortality in nulliparous and parous women aged 45 to 64 years by two categories of lifetime lactation duration (n=8,477). The HUNT2 Survey, 1995-2010.

|  |  |  | **All causes** | | | | |  | **Cardiovascular disease** | | | | |
| --- | --- | --- | --- | --- | --- | --- | --- | --- | --- | --- | --- | --- | --- |
|  |  |  |  |  |  |  |  |  |  |  |  |  |  |
|  | No. of persons | Person-years | No. of deaths | Mortality rate***^a^*** | Simple model*^b^* | Fully adjusted model*^c^* | |  | No. of deaths | Mortality rate***^a^*** | Simple model*^b^* | Fully adjusted model*^c^* | |
|  |  |  |  |  | HR | HR | 95% CI |  |  |  | HR | HR | 95% CI |
| **Women 45 - 64 years** | | | |  |  |  |  |  |  |  |  |  |  |
| *Total* | 8,477 | 119,547 | 546 | 46 |  |  |  |  | 104 | 9 |  |  |  |
| Ever lactated | 7,629 | 107,715 | 475 | 44 | 1.00 | 1.00 | Referent |  | 87 | 8 | 1.00 | 1.00 | Referent |
| Never lactated | 325 | 4,504 | 32 | 71 | 1.66 | 1.59 | 1.10, 2.28 |  | 11 | 24 | 3.14 | 3.15 | 1.66, 6.00 |
| Nulliparous | 523 | 7,328 | 39 | 53 | 1.14 | 0.87 | 0.56, 1.34 |  | 6 | 8 | 0.93 | 0.40 | 0.13, 1.26 |
|  |  |  |  |  |  |  |  |  |  |  |  |  |  |

Abbreviations: HUNT2, The second Nord-Trøndelag Health Survey; HR, hazard ratio; CI, confidence interval

***^a^*** Incidence per 100,000 person-years.

*^b^*Adjusted for maternal age.

*^c^* Adjusted for age, smoking status, physical activity, education, marital status and parity.
