# Supplementary figures and images for: A prospective population-based cohort study of lactation and cardiovascular disease mortality: the HUNT study
Source: BMC Public Health. 2013 Nov 13;13:1070. doi: 10.1186/1471-2458-13-1070 (PMC3840666; doi:10.1186/1471-2458-13-1070)

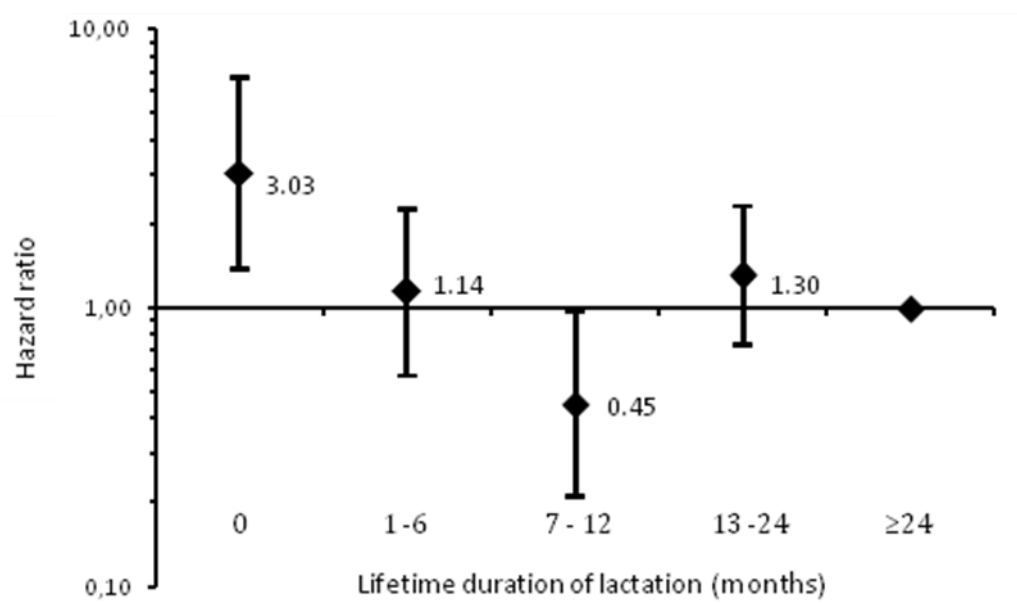

Supplement: Additional file 2: Figure S1 — Risk of death from cardiovascular disease associated with five categories of lifetime lactation duration for parous women in the age group 45 – 64 years (n = 7,954) with lifetime lactation duration ≥ 24 months as the reference category: Hazard ratios and 95% confidence intervals. Adjusted for age, smoking status, physical activity, education, marital status and parity. Number of women in each category of lifetime duration of breastfeeding, with number of deaths in brackets, 0 months: n = 325 (11), 1–6 months: n = 1,838 (22), 7–12 months: n = 2,140 (11), 13–23 months: n = 2,268 (35), ≥ 24 months: n = 1,383 (19). [file 1471-2458-13-1070-S2.pdf]
